# Low perceptual sensitivity to altered video speed in viewing a soccer match

**DOI:** 10.1038/s41598-017-15619-8

**Published:** 2017-11-13

**Authors:** Claudio de’Sperati, Gabriel Baud Bovy

**Affiliations:** 1 Laboratory of Action, Perception and Cognition, Faculty of Psychology, Vita-Salute San Raffaele University, via Olgettina 58, 20132 Milan, Italy; 2 Experimental Psychology Unit, Division of Neuroscience, San Raffaele Scientific Institute, via Olgettina 60, 20132 Milan, Italy; 3 Robotics, Brain and Cognitive Sciences Unit, Istituto Italiano di Tecnologia, via Morego 30, 16163 Genoa, Italy

## Abstract

When watching videos, our sense of reality is continuously challenged. How much can a fundamental dimension of experience such as visual flow be modified before breaking the perception of real time? Here we found a remarkable indifference to speed manipulations applied to a popular video content, a soccer match. In a condition that mimicked real-life TV watching, none of 100 naïve observers spontaneously noticed speed alterations up/down to 12%, even when asked to report motion anomalies, and showed very low sensitivity to video speed changes (Just Noticeable Difference, JND = 18%). When tested with a constant-stimuli speed discrimination task, JND was still high, though much reduced (9%). The presence of the original voice-over with compensation for pitch did not affect perceptual performance. Thus, our results document a rather broad tolerance to speed manipulations in video viewing, even under attentive scrutiny. This finding may have important implications. For example, it can validate video compression strategies based on sub-threshold temporal squeezing. This way, a soccer match can last only 80 min and still be perceived as natural. More generally, knowing the boundaries of natural speed perception may help to optimize the flow of artificial visual stimuli which increasingly surround us.

## Introduction

We are almost literally immersed in an artificial visual world, made in large part by motion pictures. How robust is our sense of reality with such pervasive technologies? For example, how well can we detect speed alterations in a video clip? The availability of videos, videogames and virtual reality systems, through which a variety of arbitrarily altered worlds can be experienced, calls for an appraisal of the boundaries within which video speed can be manipulated without conflicting with perception. However, although much effort has been devoted to studying motion perception^1,2^, how we perceive the speed of real-life scenes is still largely unknown, due especially to stimulus complexity and the presence of cognitive, higher-order factors^3–5^. Here we have addressed natural speed perception by measuring the detectability of speed alterations applied to a soccer match video clip.

## Results

### Retrospective noticing

In the first experiment we used a rather ecological setting, with observers watching a 10-minute video clip of a soccer match without any specific task. They viewed it only once and were told that at the end they would have to answer questions concerning the match, aimed to assess attention and memory capabilities. Observers were divided into five groups (N = 25 each), and each group watched the video clip at a different speed (0.88x, 0.96x, 1.00x, 1.04x and 1.12x, where 1.00x is the original video speed – 30 fps; see Methods). The original audio track with voice commentary was pitch-compensated to avoid distortions when the video was played at a different speed. In general, observers were quite compliant, as suggested by self-reported attention measures (mental concentration: 60%, event-following: 81%). By contrast, they were very poor at speed perception, as speed alterations went completely unnoticed when assessed through follow-up questions Q3 and Q4 (see Methods), aimed at evaluating the spontaneous noticing of speed change (0% accuracy in both cases), even though Q4 made reference to possible motion anomalies. It was necessary to explicitly mention speed in the question to register some correct responses (Q5, detection task, accuracy 15%). However, detection accuracy did not depart significantly from chance (P = 0.961; see Methods). This also held true when considering the four speed modifications separately (always P > 0.15). From these data, it would appear that observers almost completely neglected, in retrospect, up/down speed alterations up to ±12%.

**Figure 1:**
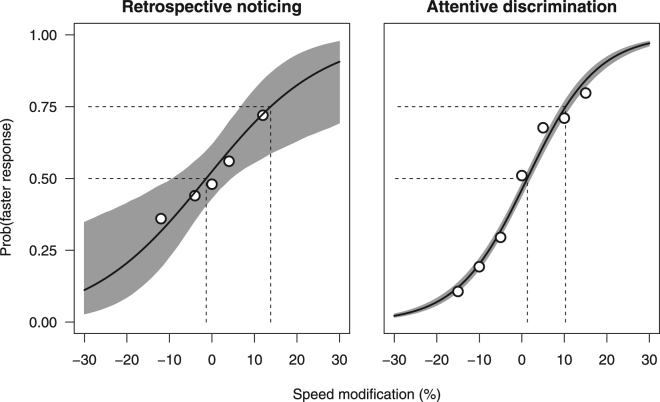
Sensitivity to video speed manipulations in the first (**A**) and second (**B**) experiment. Sensitivity was rather poor, not only in a condition mimicking ordinary TV watching (retrospective noticing, single trial testing), but also when observers were explicitly asked to pay attention to video speed (attentive discrimination, constant stimuli method), though in that case performance improved. In each plot, the black curve is the psychometric function at the population level. The grey area corresponds to the 95% confidence interval for the predicted response as a function of speed, and includes uncertainty about the fixed effects. Symbols are the proportions of observers’ ‘*faster*’ responses at each tested video speed. The vertical central line indicates the PSE (Point of Subjective Equality). The horizontal distance between the two vertical lines corresponds to the JND (Just Noticeable Difference). Speed is expressed as a percentage change to the original video speed.

To better quantify the perceptual performance in this first experiment, we fitted a standard logistic psychometric function to the responses to Q6 (which involved a two-alternative forced choice – 2AFC – response, too fast or too slow). We pooled data from all participants and used a binomial Generalized Linear Model (GLM) to fit the logistic function and parametric bootstrap to compute the 95% confidence interval of the Point of Subjective Equality (PSE) and the Just-Noticeable Difference (JND) corresponding to the psychometric function (Fig. 1A). These two quantities represent bias and sensitivity in recognizing anomalous speed at the population level, and were respectively −0.8% (95% confidence interval: −9.9–6.3%) and 17.6% (9.8–73.0%) of the original video speed. That is, observers showed a broad tolerance to speed modifications, in line with lack of evidence of spontaneous noticing and speed change detection assessed through Q3-Q5. The large confidence interval for the JND reflects the flatness of the psychometric curve. Moreover, the fact that it did not include more extreme speed alterations adds uncertainty about the exact shape of the curve. The Hosmer-Lemeshow goodness-of-fit-test was not statistically significant [χ^2^(3) = 0.332, P = 0.954], which indicates that the values predicted by the logistic function fit the data well.

### Attentive discrimination

In the second experiment we administered a psychophysical task (constant-stimuli method) involving a much larger number of observations (100 observers, 56 trials per observer). In this experiment, observers had to discriminate the speed of short segments of the soccer video clip with 2AFC responses (too fast or too slow). At variance with the first experiment, here observers had to explicitly and attentively evaluate the video reproduction speed. No standard was provided for speed comparison, thus observers had to rely on an internal model of the “natural” speed of a soccer match. To evaluate the performance at the population level, we used a binomial Generalized Linear Mixed Model (GLMM) with a logistic link function, which included a random intercept and slope for each participant^6^. The Hosmer-Lemeshow test was not statistically significant [χ^2^(5) = 2.635, P = 0.756], which indicated a good fit. We used bootstrap to compute the 95% confidence interval of the Point of Subjective Equality (PSE) and the Just-Noticeable Difference (JND) corresponding to the psychometric function. The PSE and JND turned out to be 1.2% (95% confidence interval: 0.4–2.0%) and 8.8% (8.1–9.4%) of the original video speed, respectively. The results of this analysis are shown in Fig. 1B, and were very similar to the results obtained by means of a more traditional two-level analysis, namely, GLM-based individual fitting of the psychometric functions followed by evaluation of parameter distributions (data not shown).

This pattern of results persisted even though we excluded trials with the lowest confidence (rating < 2; 94% of total trials). The PSE was 1.1% (confidence interval: 0.3–1.8%) and the JND 8.3% (7.7–8.9%), which suggests that subjective uncertainty did not affect speed judgments.

Trial order (see Methods) influenced the observers’ responses: A binomial GLMM with trial order and speed as fixed effects showed that the interaction between trial order and speed was statistically significant (z = −4.879, P < 0.001), indicating a difference of slope of the psychometric function between the two trial orders. The JND was 7.5% (95% confidence interval: 6.6–8.2%) in one direction, and 10.4% (9.5–12.1%) in the other direction. The PSEs were −0.2% (−1.2–0.5%) and 3.0% (1.8–4.2%). These differences may reflect serial dependency effects^7^ or speed adaptation^8^, which in the constant-stimuli method depend on trial history. Indeed, PSE was significantly correlated with previous speed exposure in the first experiment (P = 0.014; we recall that these observers also participated in the first experiment, where each group of participants was tested with a different video speed). Full sequence randomization (i.e., presenting a different random stimulus sequence to each observer) and/or completely crossed designs (i.e., testing all clip x speed x repetition combinations) may improve parameter estimation, but at the cost of losing the advantage of testing large subject samples in “one shot”. In both cases, however, JND was rather high.

A possible reason for the poor tuning to soccer match speed could be that, despite its popularity, observers were in fact not very familiar with soccer. Indeed, self-rated soccer expertise and soccer practice were not high (the mean soccer expertise score, SES, was 0.71 ± 0.84 S.D. and the mean soccer practice score, SPS, was 0.26 ± 0.62 S.D). However, no significant correlations emerged between JND or PSE and SES or SPS (always P > 0.1). Thus, neither generic soccer expertise nor soccer practice level seems to have affected speed judgments.

Likewise, the second experiment revealed a considerable perceptual tolerance to video speed. However, the JND was much smaller than in the first experiment, suggesting a higher sensitivity to speed. To ascertain this point, we compared the performances of the two experiments with a binomial GLMM by including the experiment as a fixed effect together with the speed. As before, the model included a random intercept and slope for the speed for each participant. The model assumed that the random effects were the same in the two experiments for those who participated in both experiments (see Methods). The Hosmer-Lemeshow test was not statistically significant [χ^2^(9) = 3.783, P = 0.925], which indicates a good fit of the combined model. Despite the large uncertainty of the shape of the psychometric function in the first experiment, this analysis revealed a statically significant interaction between the experiment and the slope of the psychometric function (z = −2.013, P = 0.044). The speed main effect was the only other statistically significant effect (z = 26.693, P < 0.001). These results confirm that observers were better at noticing video speed changes in the second experiment. Indeed, these observers explicitly focused on speed alterations, which should improve perceptual performance.

A possible confounding factor is that we used a custom program to deliver the video sequence with the sound muted in the second experiment, whereas in the first experiment an open-source video player with pitch-compensated original audio-track was used (see Methods). Although the basic algorithm for speed change was identical in the two software solutions, we could not exclude subtle differences in motion rendering; also, the lack of audio-track may have changed visual sensitivity to anomalous motion in the second experiment. Therefore, we ran a control experiment to assess the impact of the different audio-visual conditions. More specifically, we repeated the second experiment (constant stimuli), but using the same setup as in the first experiment, including pitch-compensated audio-track (and with 25 participants instead of 100, 56 trials per subject). The results were very similar to those obtained in the second experiment, with PSE = 1.5% (95% confidence interval: 0.3–2.7%) and JND = 9.9% (8.8–11.2%). A direct comparison between the two experiments via a GLMM including experiment and speed as fixed effects did not reveal any statistically significant effect associated with the experiment or with the interaction between the experiment and the slope of the psychometric function (z = 0.004, P = 0.997, and z = −1.020, P = 0.308, respectively). Only the speed factor was statistically significant (z = 28.5, P < 0.001). Thus, there was no evidence that either subtle differences in motion rendering or the audio track affected the sense of speed in any important way.

## Discussion

The results of this study documented a poor sense of speed in soccer viewing. Firstly, in retrospect participants completely missed anomalous video speeds with modifications of up/down to 12% (first experiment, Q3-Q5). For comparison, consider that in experiments on inattentional blindness, where unattended elements in a visual scene, even important ones, are neglected, noticers are often a non-negligible proportion – sometimes the majority – of observers^9^. Indeed, in the first experiment JND estimates (Q6) were quite large (∼18%). In the second experiment, when observers were explicitly asked to pay attention to speed alterations, JND was still high, although much lower than in the first experiment (∼9%). This figure did not change significantly in the control experiment, which suggests that using different software solutions or muting videos did not affect the sense of speed, at least in our experimental conditions.

Our JND estimates are generally consistent with figures obtained under quite different laboratory conditions, stimuli and paradigms in humans^10,11^ and in monkeys^12^, as well as with more or less realistic optic flow stimuli in car driving contexts^13–16^. However, we also directly compared the results obtained in a classical laboratory setting (second experiment) with those obtained in a condition that mimicked home TV watching (first experiment), showing in the latter case an even poorer sensitivity to altered video speed. Indeed, in the first experiment observers’ attention was focused on the match and not on video speed, a condition that is expected to lower sensitivity to anomalous speeds. This result underscores the importance of pairing structured psychophysical designs with tasks for retrospective noticing in an ecological condition, at the cost of “burning” participants after the critical trial, which requires a large number of participants^9,17^. Clearly, the merit of the first experiment is also its weakness: in order to mimic real-life conditions observers should be naïve, but in order to be naïve they should be tested only once. Hence, to reach definitive conclusions on real-life conditions, a very large-scale study would be required, targeting also the relevant population characteristics (e.g., different ages, media consumption habits, viewing environments, etc.). Still, the fact that important speed modifications went completely neglected is remarkable. By leveraging on our JND estimates, and keeping in mind the limits of approximate estimations in the first experiment, we suggest that speed tolerance in real-life conditions can be extrapolated to be about double of that measured with the constant stimuli method.

As to the small but significant speed underestimation found with the constant stimuli method, indexed by PSE, various factors may be at play. For example, speed judgments may be influenced by the brain’s perceptual tuning to low visual speeds, a bias that has been posited to emerge under uncertainty conditions with weak, low-contrast stimuli^10,18–20^. Videos are indeed low-contrast, reduced versions of real scenes. Other explanations are possible, however, based on distortions of temporal perception with motion stimuli^3^, intuitive physics biases^5^, or motion anticipation tendencies^21^. Violation of speed constancy could also account for speed underestimation^22,23^.

Interestingly, our study justifies the practice of playing 24 fps movies at 25 fps in PAL systems. Indeed, the frame rate difference (4%) would be largely sub-threshold, even if observers were to focus on speed rather than the film plot. Slightly different is the case of some old silent films that have an evident jerky appearance, which indicates that the mismatch between footage and reproducing speeds was sometimes quite large^24^. Such a large mismatch may have been partly intentional, as both camera operators and projectionists contributed to the cinematographic language by manipulating speed^25^. Indeed, altering the reproduction speed can even influence intent attribution^26^. At any rate, explicit speed modifications, including special effects such as instant replay, fast-motion or time lapse break our sense of reality and thus are definitely different from the sub-threshold speed changes targeted in the present study, which preserve the illusion of authenticity and a subjectively normal event flow.

Clearly, the present findings cannot be generalized, as they apply to a specific visual content, though a very common one. It is possible that speed tuning improves when more definite motion cues are available, e.g., close-up of human movements (which might activate specific visual or visuo-motor cortical circuits to detect anomalous movements or biomechanical inconsistencies^9,27–30^), or apparent violations of physical laws (e.g., if a heavy object exhibits little inertia). By contrast, it is likely that speed tolerance gets worse for more kinematically-ambiguous visual events such as water flow. This remains to be tested.

An obvious consequence of a poor sense of video speed is that editors and broadcasters – as well as individual users – can implement compression by speeding up videos, thus freeing up viewers’ time. The need to keep speed alteration sub-threshold to preserve the perception of normality would limit the range of possible manipulations. However, even a small speed correction would have a strong socio-economic impact. Speeding up an important soccer match by 10% could yield an enormous monetary gain, if the time saved is replaced with ads. Similarly, a shortened ad would cost less. The possibility that speed sensitivity depends on the visual scene (e.g., close-ups of human movement or ambiguous scenes) suggests that compression should not be applied uniformly, though. For example, to remain in the domain of soccer, the normal actions shot with a very wide angle may undergo stronger compression than special camera shots. How precisely compression should be implemented in order to avoid breaking the illusion of natural speed is a matter for future investigation.

Before applying speed-based compression indiscriminately, however, it will be important to appraise possible side-effects. For example, long-term exposure to high video speeds may lead to perceptual adaptation^8^, and possibly even subtle addiction phenomena^31^. If liberalizing speed is the future of video technologies, we should be prepared for sub-threshold but intensive “kinetic hyper-stimulation”. Measuring speed tuning to natural speeds, in both attentive and inattentive conditions, would be a prime monitoring tool.

## Methods

### Participants

We recruited 125 students enrolled in our Psychology study program (105 females, mean age = 22.0, naïve to the purpose of the experiment). They took part in the first experiment and subsequently in the second or the control experiment on a voluntary basis, and gave informed consent prior to the beginning of the experiments. The study was conducted in accordance to the principles of the Declaration of Helsinki and the San Raffaele Ethical Committee.

### Procedure

In the first experiment, participants were assigned to 5 groups (N = 25 each), and were blind to experimental manipulations. Participants sat in a classroom and watched a 10-min soccer video clip, in separate sessions. Each group watched the video clip played at a different speed: 0.88x, 0.96x, 1.00x, 1.04x or 1.12x. The group with speed = 1.00x consisted in fact of 2 subgroups tested in separate sessions (see below). At the end, the experimenter verbally asked the observers the following questions one by one, which they responded to in writing.

Q1: Rate your concentration during the task (0–10)

Q2: Which team had more ball possession?

Q3: Did you notice anything strange in the video? If so, provide a brief description.

Q4: Did you notice anything strange in the colour, shape or motion of the video? If so, provide a brief description.

Q5: Was the video too slow/was the video too fast?

Q6: Was the video too slow or too fast?

Questions 1 and 2 served as generic indications of how much attention observers paid to the match, and targeted mental concentration and event following, respectively. Questions 3 and 4 quantified spontaneous noticing, the latter also providing three cues, one regarding motion. Question 5 configured a yes/no detection task, and consisted of 2 versions (see below). Question 6 configured a 2AFC discrimination task, where symmetry of the responses discourages bias. In passing from Q3 to Q6, the idea was to add specificity to each subsequent question. Thus, the Q3-Q6 sequence can be viewed as a progressively more stringent probing tool. Similar questions appear in commonly adopted protocols for subjective video quality assessment (e.g., standard ITU-T P.910). Note that responding to Q6 is not subjected to the bias that might be present with Q3-Q5, which would manifest as a tendency to prefer the “no” response. Any bias with Q6would instead manifest as a preference for responding “too slow” or “too fast”, thus affecting PSE.

In the second and the control experiments, participants (N = 100 and N = 25, respectively) seated in a classroom watched a sequence of short segments of the same soccer video clip, whose reproduction speed was manipulated (7 speeds, from −15% to 15% in 5% steps, see below). They had to report on paper, trial by trial, whether speed appeared increased or decreased compared to the “natural”, original version, which was however never shown as standard (constant-stimuli method, one-interval 2AFC discrimination task), as well as a confidence rating for the response (in a 0–10 scale). Note that the 2AFC question was identical to Q6 of the first experiment. In the second experiment, half of the participants were presented a randomly generated stimulus sequence, and the other half viewed the same sequence in reverse order, in different sessions. For the control experiment, which had fewer observers, a third video sequence was used.

Before the beginning of the experimental sessions, participants self-rated their soccer expertise and soccer practice levels on a 0–3 scale (0 = scarce or null, 1 = fair, 2 = good, 3 = excellent). These scores were normalized and expressed as percentages.

### Stimuli and apparatus

The video clip was an accredited recording of an international soccer match, including the original soundtrack with voice commentary. A short 1280×720@30 fps version was obtained through video editing, lasting 9′36″. For simplicity we refer to it as to the 10-minute or short version (readers can contact the author to access the video clip). Editing also served to exclude irrelevant parts of the original video (e.g., slow-motion replays of important actions, or zooms on details such as coach or public, etc.), and to place a grey patch over two frame regions containing an on-screen advertisement and the timing of the match, which could be a cue to recovering the true speed. This edited version was used in the first experiment. For the second and the control experiments, we randomly extracted 56 segments from the edited version, each lasting 15 seconds, one for each trial.

For video clip reproduction, the open-source program VLC (https://wiki.videolan.org/) was used in the first and the control experiments, while in the second experiment a custom program was written in Matlab, using the Psychophysics Toolbox extensions. In the first experiment, we selected 5 speeds through VLC controls, nominally corresponding to speed gains of 0.88x, 0.96x, 1.00x, 1.04x and 1.12x. By comparing the true match duration (visible on the superimposed match timing) and the actual duration measured with a stopwatch in each speed condition, we empirically derived the effective video speeds achieved in the first experiment, which turned out to be 26.49, 28.81, 29.97, 31.18 and 33.87 fps, corresponding to 0.88x, 0.96x, 1x, 1.04x and 1.13x. For simplicity, we refer to this range as ±12%, and to the original speed as 30 fps. Given that calibration was performed over the entire 10-minute clip, and that stopwatch reading uncertainty can be estimated to be no more than 0.5 s, the measurement error was likely well below 0.1%. In the second experiment, we directly controlled video frame flipping, and dissociated the frame rate from the refresh rate (which was fixed at 60 fps in all experiments), that is, we retained a frame for a number of refresh cycles, depending on the desired video speed. This way, we avoided two visual artefacts, namely, video tearing, which would have resulted from a crude disabling of the V-sync signal and unnatural motion, which could have resulted from frame interpolation. The frame rates achieved were 25.51, 27.00, 28.50, 30.00, 31.47, 32.97 and 34.50 fps, corresponding to speed ratios of 0.85x, 0.90x, 0.95x, 1.00x, 1.05x, 1.10x and 1.15x, thus in a range very similar to that used in the first experiment. Note that VLC does not actually implement frame interpolation and that video frame flipping is generated with the same principle that we used in the custom Matlab program, i.e., dissociating the frame rate from the refresh rate (J.B. Kempf, personal communication). For all experiments, a Sony fhz57 video-projector was used, driven by a Sony Vaio z equipped with an Nvidia GeForce GT330M graphic board, with V-sync enabled. The video clip was projected on a 100″ wall screen. The final projection quality was quite good, as attested by the near lack of observers’ remarks about video quality (only 5% of them reported anomalies at either Q3 or Q4 other than speed changes, e.g., flickering or unnatural colours).

### Data analyses

In the first experiment, mental concentration during the task was self-rated in a 0–10 scale (Q1), while event-following was assessed through correct responses to Q2, both expressed as percentages. For spontaneous noticing (Q3 and Q4), only responses containing a reference to speed counted as positive evidence (correct responses).

Detection accuracy was computed from Q5, and was defined as the ratio between the number of noticers (response “Yes!”) and the number of observers who watched the video at an altered speed. Note that question Q5 actually had 2 versions, one posed to those observers who watched a low-speed video (“Was the video too slow?”), and the other posed to those observers who a watched high-speed video (“Was the video too fast?”). This way, the task retained a yes/no detection and not a 2AFC discrimination structure. This also held for observers who watched the video clip at original speed, which were divided into two subgroups, each receiving the question in one of the two versions. We first computed a global false alarm rate, defined as the proportion of “noticers” (response “Yes!” to either question version) among those observers who watched the video clip at the original speed, regardless of the question version. The false alarm rate was also computed separately for the two question versions, which served as the correct comparison term when accuracy was measured for each individual observer’s group (with the former version being used when the video was slower, and the latter when the video was faster). False alarm rates were therefore the chance level for evaluating the detection performance with the χ^2^ test.

In the first experiment where a single response was obtained for each subject, we pooled data from all participants and fitted a logistic function with speed as predictor. To that end, we used R *glm* function. For the constant stimuli experiments, we used a GLMM approach to fit an individual psychometric curve to all subjects simultaneously^6^. To that end, we used the *glmer* function from the *lme4* R package. In our analyses, all GLMMs included the intercept and speed in the fixed and random effects. In this framework, fixed effects represent the general tendency at the population level while random effects represent the individual deviations from the general trend. The random effects are assumed to follow a multivariate normal distribution with mean zero and unstructured variance-covariance matrix. The data did not show signs of over-dispersion, as indicated by the ratio between the Pearson residuals sum of squares and the residuals degrees-of-freedom, which was less than 1.1 in all experiments. To compare trial order in Experiment 2 or the psychometric functions across experiments, we included the corresponding factors among the fixed effects. We used the Wald test to compute the P values and parametric bootstrap with the percentile method to compute the confidence intervals (N = 500)^32,33^. Goodness of fit for GLM and GLMM models was assessed with the Hosmer-Lemeshow test, using the number of speed levels to group the data^34^.

The Point of Subjective Equality (PSE) was defined as the video speed at which an observer judged a speed increase or decrease as equally probable (corresponding to 50% of the psychometric function). The Just-Noticeable Difference (JND) was defined as the semi-inter-quartile difference of the psychometric function. Both PSE and JND are expressed as fractions of the original frame rate (30 fps). JND is always positive (which implies that for a JND of, say, 10%, the upper and lower thresholds are at +10% and −10% from PSE, respectively), while PSE is a signed value. PSE and JND were computed with the fixed effect parameters of the fitted models: PSE = −β_0_/β_1_ and JND = log(3)/β_1_ where β_0_ is the intercept and β_1_ the slope of the speed factor.

### Availability of materials and data

The datasets generated and/or analysed during the current study are available from the corresponding author.
